# Noncredible Complaints and Symptom Validity in Patients With Chronic Pain

**DOI:** 10.1155/prm/7422265

**Published:** 2025-09-23

**Authors:** Jared G. Smith, Linda Monaci, Martin D. van den Broek

**Affiliations:** ^1^St George's School of Health and Medical Sciences, City St. George's University of London, London SW17 0RE, UK; ^2^Clinical Neuropsychology Private Practice, Surrey KT18 7PF, UK; ^3^The Neuropsychology Clinic, Parallel House, Guildford GU1 2AB, UK

**Keywords:** chronic pain, malingering, noncredible, over-reporting, personal problems questionnaire, symptom validity

## Abstract

**Introduction:** The multifactorial nature of pain complicates assessment of the validity of presenting symptoms and behaviours in people with chronic pain. Recently, the Personal Problems Questionnaire (PPQ) was developed to assess genuine and noncredible cognitive, emotional and physical complaints. Here, the PPQ was used to investigate the extent to which patients with chronic pain report noncredible complaints and the relationship with pain severity and measures of cognitive performance validity and symptom over-reporting.

**Materials and Methods:** Seventy-five participants with chronic pain recruited from outpatient and pain management programme clinics completed the clinical and validity scales of the PPQ, the short-form McGill Pain Questionnaire (SF-MPQ) subscales and the Medical Symptom Validity Test (MSVT), and a subsample (*n* = 27) completed the Personality Assessment Inventory (PAI).

**Results:** Significant mean (T-score±SD) elevations were observed across the PPQ cognitive (64.5 ± 13.1), emotional (65.1 ± 13.2) and physical (77.4 ± 11.0) clinical domains. Endorsement of implausible complaints on the PPQ was common; 35.6% of patients endorsed noncredible pain/physical complaints, while 19.2% and 33.3%, respectively, reported implausible cognitive and emotional difficulties. Multivariate analyses indicated that the odds of likely noncredible responding significantly increased in cognitive (34%) and emotional domains (26%) and in the physical domain (12%) for every point increase on the SF-MPQ affective and sensory pain subscales, respectively. Noncredible symptom reporting was elevated in those receiving disability benefits/involved in litigation (*n* = 27), but not significantly after controlling for pain severity. Negative impression management on the PAI was associated with implausible cognitive and emotional symptom endorsement, but there was a limited relationship between PPQ validity scales and MSVT underperformance.

**Conclusion:** The PPQ is a potentially useful tool in the assessment of chronic pain patients, with implausible symptom endorsement found in a significant proportion, although this may not reflect intentional exaggeration.

## 1. Introduction

Chronic pain is frequently associated with limitations affecting the individual's quality of life [1, 2]. In many cases, sufferers undergo a progressive deterioration in function with associated movement restrictions and learnt inactivity, as well as changes in sleep and appetite, and a risk of dependency on medication [3, 4]. Mood disturbances also frequently occur, such as anxiety, depression and somatic preoccupations [5, 6], and neurocognitive abnormalities can develop, most commonly revealed via tests of memory, attention and processing speed [7]. Some people with chronic pain become unable to work and have difficulty maintaining social interactions, leading to a progressively restricted life [5, 8], and, in some instances, due to loss of income, become reliant on insurers and/or state benefits for financial support [9].

Pain is usually considered to be chronic when it persists for 3–6 months without resolution, extending beyond normal tissue healing time [10]. While it is common, affecting approximately 20% of the population [11, 12], there have been concerns that some individuals with pain-related disability may be nongenuine and involve malingering [13]. For instance, in an older survey study, Mittenberg, Patton, Canyock and Condit [14] found that malingering was estimated to occur in 31% of those with chronic pain, and Greve, Ord, Bianchini and Curtis [15] concluded that malingering was present in 20%–50% of those seen in a medico-legal context.

The factors driving complaining may be difficult to discern. Etherton [16] distinguished between incentive effects that arise from involvement in a disability claim and the prospects of financial compensation, and tertiary gains that accrue to other individuals, such as physicians, mental health workers and the legal profession, who may be incentivised by payment for treatment or legal services. In these circumstances, rapid recovery may lead to the cessation of payments and greater disability increases the amount earned by legal advisers, leading to prolonged disability. However, even in medico-legal claims, the incentive may not be financial, but relate to other potential gains, such as avoiding legal penalties or the provision of opiate medication which can be resold. On occasions, the gains may be obscure to the treating clinician [17, 18]. However, while some may exaggerate or fabricate pain complaints, it may not be due to malingering but rather to satisfy an internal need, such as in the case of factitious disorder or pain associated with somatisation.

Tuck, Johnson and Bean [19] outlined four strategies that have been used to detect nonvalid pain complaints in people with chronic pain: behavioural signs, effort measures, questionnaires and performance validity tests (PVTs). Although overt pain behaviours, such as moaning, sighing and bracing, are often thought to be exaggerated, these illness behaviours have not been found to be reliably associated with deliberate deception, and Tuck et al. [19] suggested that they may instead reflect the result of operant conditioning. The use of physical effort measures has also been found to be wanting, whereas multidimensional questionnaires, such as the Personality Assessment Inventory (PAI [20]) and Minnesota Multiphasic Personality Inventory-2-RF (MMPI-2-RF) [21], have proved more promising. Aguerrevere and colleagues [22] examined a forensic group of pain patients who had external incentives (i.e., compensation claims) and found over-reporting on the MMPI-2-RF validity indicators together with elevations on the restructured clinical scales differentiated between genuine and nonvalid pain complaints. In recent years, PVTs such as the Test of Memory Malingering [23] and the Word Memory Test [24] have also been used in pain assessments. Although cognitive measures do not directly assess the validity of individuals' reports of pain, many patients with chronic pain have complaints crossing several domains, including concentration and memory, and therefore, it is arguably appropriate to utilise neuropsychological measures in conjunction with specific pain measures. The development of criteria for malingered pain-related disability (MPRD), which utilises PVTs [25], has reinforced their use in clinical practice.

A difficulty with multidimensional questionnaires, such as the MMPI-2-RF, is that it may not be clear whether elevations found on validity scales reflect exaggeration or genuine and severe psychopathology [26]. This arises because such scales typically comprise genuine complaints and questionable validity is inferred when an individual endorses an excessive number, rather than the complaints themselves being implausible. A different approach was adopted by van den Broek et al. [27] who developed the Personal Problems Questionnaire (PPQ). The PPQ includes validity scales involving items that are a priori noncredible as they involve implausible or overly specified symptoms, overly severe complaints or unusual symptom combinations. The validity scales assess physical, cognitive and emotional complaints, and van den Broek et al. [27] found that they showed satisfactory sensitivity and specificity in differentiating between healthy individuals, neurological patients and those simulating neurological impairment. An important advantage of such a measure is that over-reporting on the validity measures is inherently implausible, so allowing the clinician to more confidently conclude that an individual's presentation is noncredible.

The aim of the present study was to investigate self-reported credibility on the PPQ in those with chronic pain. In addition, the PPQ provides the opportunity of determining the extent to which noncredible pain and physical complaints are associated with a comparable tendency to implausible symptom presentation in domains relating to cognitive and emotional complaints. Finally, the study examined the degree to which noncredible self-report on the PPQ is associated with experienced pain intensity levels, invalid responding on another symptom validity test (SVT), the PAI [20], a PVT and the Medical Symptom Validity Test (MSVT) [28]. Previous research has suggested that while SVT and PVT measures are associated, and those who fail effort tests may also over-endorse symptoms, this is not an invariant finding and symptom over-reporting and underperformance can be separate constructs [29, 30]. The current investigation therefore sought to determine the extent to which implausible responding on the PPQ was associated with these measures in patients with chronic pain.

## 2. Materials and Methods

### 2.1. Participants

Seventy-seven patients with chronic pain were recruited to the study drawn from a hospital-based outpatient chronic pain service and a day-patient pain management programme (PMP). Patients were diagnosed as having chronic pain by a medical pain consultant and members of the pain management multidisciplinary team. Those attending the outpatient clinic were referred from primary care and secondary care specialists (e.g., orthopaedic surgeons). The PMP was intended for patients for whom medical management had been unsuccessful and a cognitive behaviour therapy (CBT)–based psychosocial approach was thought to be beneficial. Patients participated in the PMP following assessment by a multidisciplinary staff group. Participants were recruited via consecutive, opportunistic sampling. Written informed consent was provided by all participants. This study was granted ethical approved by the Wandsworth National Research Ethic Service and the Faculty Ethics Committee at the University of Essex and government approval by the Research and Development Department of St George's University Hospitals NHS Foundation Trust.

All study participants met the following criteria: (a) age ≥ 18 years; (b) experiencing chronic pain (i.e., pain ≥ 6 months); and (c) basic standard of reading and understanding in English assessed through initial interview and discussion with the medical consultant (outpatient clinic) or multidisciplinary team (PMP); (d) absence of acquired or degenerative brain dysfunction or disease expected to interfere with questionnaire completion as identified by medical records; and (e) were not actively psychotic, suicidal and/or having misused alcohol or illicit drugs in the 3 months prior to study (as indicated by their treating Medical Consultant (outpatient clinic) or multidisciplinary team [PMP]).

### 2.2. Measures

Sociodemographic data, including employment status and whether participants were receiving disability benefits or undergoing litigation-related determinations, were collected. Participants were assigned to 3 educational attainment groups according to the following criteria: low = no qualifications; medium = GCSEs, A-levels (or other US equivalent General Educational Development/Advanced Placement credential); high = college-level diploma or degree. The following questionnaires were administered:

#### 2.2.1. Short-Form McGill Pain Questionnaire (SF-MPQ) [31]

The SF-MPQ was administered to assess the sensory and affective dimensions of pain in the study sample. This tool comprises 15 pain descriptors (11 sensory; 4 affective), each of which is rated on a four-point intensity scale (0 = none, 1 = mild, 2 = moderate, 3 = severe). Summative scores for sensory, affective and total descriptors were calculated by summing the scale responses on the relevant items (potential score range = 0–33, 0–12 and 0–45, respectively). The internal consistency of the SF-MPQ scale (*α* = 0.89) and sensory (*α* = 0.85) and affective (*α* = 0.79) subscales in the study sample was satisfactory.

#### 2.2.2. PPQ

The PPQ is a self-report questionnaire, which was developed to assess cognitive, emotional and physical symptoms, and incorporates validity scales for each of these domains. It consists of 156 questions relating to symptoms commonly observed in those with chronic pain and other acquired conditions. It comprises 12 clinical subscales of 10 items each, and 3 validity scales of 12 items each, and a response consistency scale. Each of the 12 clinical subscales belongs to one of three domains (cognitive, emotional, physical), and the four subscales in each domain are summed to produce three total clinical scales, with high scores indicating increased levels of symptomatology. The 3 validity scales correspond to each of the 3 total clinical scales. The validity scale items were designed to represent implausible complaints, overly specified or overly severe symptoms or unusual symptom combinations that would not be endorsed by healthy individuals or those with a clinical condition. To maintain test, security sample items are not included, but to illustrate they include questions such as whether certain sounds affect the individual's memory (implausible cognitive complaints) or whether their hearing was affected by feeling low (implausible physical symptom combination). Subjects are asked to read each question and rate the extent to which they have experienced the problem on a three-point scale (Never = 0, Sometimes = 1, Often = 2) during the preceding month. The instructions indicate that the questionnaire is not a test and there are no right or wrong answers and respondents should ensure that all questions are answered.

Scores on each of the clinical subscales range from 0–20. A total clinical score for each domain (total cognitive, total emotional, total physical) can be derived from the 4 subscales within each domain, ranging from 0–80. In addition, domain-specific T-scores can be calculated based on the difference between the observed and demographically predicted total clinical scores, the latter derived from the regression-based norming procedure (with a mean T-score of 50 and SD of 10, accounting for age, gender and education level) described by van den Broek et al. [32] Raw scores on the 3 validity scales range from 0–24, with “likely noncredible” classifications for each validity scale made at score ≥ 5 for individuals with medium–high education levels and ≥ 8 for those with low education levels, in accordance with recommended cut-offs in the normative study [32]. The response consistency scale checks that 9 semantically similar items are responded to in a similar manner, with T-score > 80 (+3 SD over the mean based on normative population scores) [32] indicating excessive variable response inconsistency, such that the results should not be interpreted.

#### 2.2.3. MSVT [28]

The MSVT is a brief verbal PVT designed to assess cognitive effort and memory. The MSVT displays 10 common word pairs over two consecutive trials; measures of immediate recognition per cent correct (IR%) and delayed recognition per cent correct (DR%) were administered after a 10-min delay and consistency between the two scores (CNS%) was calculated. The standard cut-off score of < 85% in each of the recognition indices was utilised to indicate a failure of effort. This cut-off reflects performance at least two SDs below the mean for adults and children responding consistently [28] and has yielded 100% sensitivity and specificity between simulators and a control group in an analogue study of experimental malingerers [33].

#### 2.2.4. PAI [20]

The PAI is a multidimensional self-report measure comprising 344 items and 22 nonoverlapping scales. The clinical scales relate to mood issues, substance use and treatment. The PAI also includes 2 validity scales, which were the measures reported on here: negative impression management (NIM), comprising exaggerated or bizarre symptoms and positive impression management (PIM), which addresses the tendency for individuals to present themselves in a favourable light. Each validity scale has a cut-off score differentiating between ‘valid' and ‘moderately/severely distorted' responding (NIM > 73; PIM > 56) [20].

### 2.3. Procedure

Patients recruited through the pain clinics and the PMP were offered participation on the day (after their appointment or group session, respectively) or an appointment at a later date. Participants were informed that the study was interested in their experiences of symptoms for the purpose of evaluating the PPQ with chronic pain patients. In common with similar research on symptom validity, full information and debriefing about the project aims was not provided so as not to invalidate the study and potentially impact on the therapeutic relationship with staff based in the service that hosted the research. However, in the event that a participant scored in a clinical range on the PAI suicidal ideation scale, their treating psychologist or physician was informed.

After each patient was consented to the project, they completed the PPQ, followed by the MSVT immediate recall and a delayed recognition trial. Due to the length of time needed to complete the PAI, participants were given the option of taking part in either the ‘short' or the ‘long' version of the study. The short version included the PPQ and MSVT, and the long version additionally included the PAI. Only a subsample of patients (*n* =* *27) opted for the ‘long' version and completed the PAI.

### 2.4. Statistical Analysis

Descriptive data were summarised using mean (SD), median (interquartile (IQR) range) and frequency (%) according to data distribution. Generalised linear models (GLMs) with linear and logistic model types were used to examine factors associated with PPQ clinical scale *T*-scores and likely noncredible classifications on the validity scales. Factors included pain duration (years), sensory and affective pain severity scores, and a dichotomising variable indicating whether or not patients were potentially financially incentivised (involved with litigation and/or receiving disability benefits). Only variables showing significant associations (i.e., *p* < 0.05) in univariate analyses (Pearson *r*, Spearman *rho* and independent group *t*-tests with bootstrapping where continuous distributions did not approximate a normal distribution) were included in GLM. The quadratic function of age was included in GLMs for emotional and cognitive validity scale classifications (given the observed relationships in the normative study of van den Broek et al.) [32], although the corresponding coefficients were not reported. Magnitudes of effects in linear and logistic GLMs were described by unstandardised beta (B) values and odds ratios (OR), respectively, with 95% confidence intervals (CIs). Sensitivity and specificity values and positive predictive power (PPP) and negative predictive power (NPP) were calculated to assess the extent to which likely noncredible responding on the PPQ was associated with MSVT failure and invalid responding on the PIM and NIM subscales of the PAI. Statistical analyses were carried out using SPSS (IBM, Version 28.0) with a criterion for statistical significance of *p* < 0.05.

## 3. Results

The sociodemographic and clinical characteristics of the sample are shown in Table 1. Almost two-thirds were female, with more than 40% highly educated. Almost 40% of patients were working or studying, while about half were receiving disability benefits or medically retired. Most patients had been experiencing pain for more than 3 years, and SF-MPQ scores indicated moderate levels of pain on average. There were no significant differences between patients recruited from the clinic versus those participating in a PMP with respect to demographic variables, whether they were receiving disability benefits or involved in litigation, and SF-MPQ pain severity (for all comparisons, *p* > 0.066).

### 3.1. PPQ Clinical and Validity Scales

As a group, chronic pain participants showed acceptable response consistency; the mean score on the response consistency index (potential range 0–9) was 3.44 (SD = 2.12) with 87% of scores between 0 and 5. Two chronic pain participants had inconsistency scores above the 80T threshold used to define inconsistent responding [32]; these patients were excluded from subsequent analyses.

Table 2 contains the summary statistics for the participants on the PPQ clinical and validity scales. Clinical scales and subtests and validity scales all showed adequate internal consistency of comprising items (*α* ≥ 0.75). Scores on clinical subscales were, generally speaking, wide-ranging in the chronic pain sample; all means were markedly higher than those observed in the community-based participants in the normative study (where mean (SD) scores ranged from 1.34 (2.00) for disability and restrictions to 4.41 (3.40) for anger). The mean T-score for the total physical scale was elevated by almost 3 SDs, this being consistent with the expectation that those with chronic pain endorse significantly greater pain-related complaints, somatic complaints and restrictions. However, significant mean elevations (65T [32]) were also found for both the total cognitive and emotional scales, indicating that the participants' complaints crossed domains. Scores on scales were highly interrelated; total physical scale T-scores were moderately associated with both total cognitive (*r* = 0.76, *p* < 0.001) and total emotional (*r* = 0.76, *p* < 0.001) scale T-scores; the latter were themselves closely linked (*r* = 0.86, *p* < 0.001).

**Figure 1:**
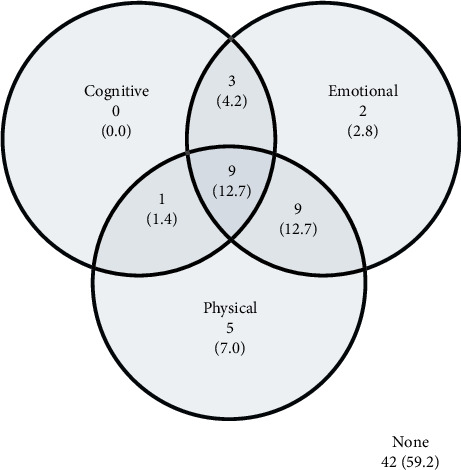
Venn diagram showing frequency (%) of patients in each domain showing likely noncredible reporting on the PPQ (*n* = 71). Please note that only patients with scores on all 3 validity scale domains are included.

Mean validity scale scores were low, with almost 60% of patients scoring 2 or less on the cognitive (58.9%) and emotional validity scales (59.7%) and a little under half doing so on the physical (47.9%) validity scale (Table 2). Elevations indicating high levels of implausible responding were found in about one in five (19.2%) patients on the cognitive validity scale, one in three (33.3%) on the physical validity scale, and 35.6% on the emotional validity scale, however. Patients tended to report implausible pain-related symptoms on the physical validity scale as occurring ‘Sometimes' or ‘Frequently' (Mean = 33.2%, SD = 31.5) more often than other (non–pain-related) implausible physical symptoms (Mean = 19.5%, SD = 22.3; *p* < 0.001). As noted previously, likely noncredible classification thresholds are adjusted for education level, and (with this adjustment) there was no indication that patients with low education levels were more likely to exceed these thresholds than those with medium–high education levels (cognitive validity scale: 20.0% vs. 19.0%, *p*=0.999; emotional validity scale: 40.0% vs. 31.6%, *p*=0.538; physical validity scale: 26.7% vs. 37.9%, *p*=0.550). Of those patients evidencing implausible responding in any domain, most did so in more than one domain (Figure 1).

### 3.2. Impact of Pain Severity and Disability Benefit/Litigation Status on PPQ Scale Responses

Univariate analyses showed that increasing sensory and affective pain severity and receiving disability benefits and/or litigation involvement were significantly linked to higher *T*-scores on cognitive, emotional and physical clinical scales (Table 3). Although multivariate analyses indicated that affective pain severity significantly contributed to variance in *T*-scores on all clinical scales, sensory pain severity was independently associated with physical clinical scale *T*-scores only, while disability benefit/litigation status was independently related to cognitive clinical scale *T*-scores only.

On cognitive, emotional and physical validity scales, mean sensory and affective pain dimension scores were significantly elevated in individuals with high levels of implausible responding (Table 4). Those patients receiving disability benefits and/or involved in litigation were also more frequently classified as likely noncredible responders in cognitive and emotional domains. GLM analyses indicated that the odds of likely noncredible responding in cognitive and emotional domains, respectively, increased by 34% and 26% for every point increase on the SF-MPQ affective pain subscale and in the physical domain increased by 12% for every point increase on the SF-MPQ sensory pain subscale. Receiving disability benefits and/or litigation involvement more than doubled the odds of likely noncredible responding on both cognitive and emotional validity scales, although after controlling for pain severity, these effects were no longer significant.

### 3.3. Implausible Responding on the PPQ, MSVT Failure and Distorted Responding on the PIM and NIM Subscales of the PAI

The number of participants who failed the MSVT on the basis of conventional cut-offs (i.e., ≤ 85%) on the recognition or consistency measure and evidenced moderately or severely distorted responding on the PAI PIM and PAI NIM subscales are shown in Table 5, together with those who scored above or below the PPQ validity cut-off scores. The MSVT and PPQ tended to have better NPP than PPP, indicating that valid scores on the two measures tended to concur, but the sensitivity of the PPQ validity scales to MSVT failure was modest. Relative to the NIM scale, the PPQ cognitive and emotional validity scales both had satisfactory specificity, and their sensitivity was also satisfactory (i.e., > 90%). The sensitivity of the PPQ physical validity scale was good, whereas the specificity was relatively weak (i.e., < 90%). In the case of the PIM scale, all of the PPQ validity measures had unsatisfactory specificity and sensitivity.

## 4. Discussion

The aim of the present investigation was to assess the extent to which noncredible reporting occurs in patients with chronic pain. While using self-report on questionnaires is not novel, the predominant approach taken to date has been to evaluate the extent to which genuine symptoms are endorsed to an excessive degree, with the risk that inferences about exaggeration may be erroneous because such reporting may simply reflect genuine or severe psychopathology [19]. In contrast, the PPQ includes clinical scales which comprise items that address a wide range of plausible complaints (cognitive, emotional and physical) and (corresponding) items that involve symptoms and symptom combinations that are a priori implausible, with high levels of endorsement for the latter suggestive of noncredible symptom presentation. The findings showed that patients with chronic pain endorsed having high levels of (credible) pain and physical complaints, approaching 3 SD above (population) norms after adjusting for age and educational level. Patients also reported marked cognitive and emotional impairment, which was linked to higher (affective) pain intensity levels, consistent with the known relationship between chronic pain and maladaptive neurocognitive and emotional function [4, 7, 34]. However, this was accompanied by high levels of noncredible symptom reporting on the validity scales, with 35.6% reporting implausible physical complaints, and 19.2% and 33.3% providing noncredible symptom endorsement on the cognitive and emotional validity scales, respectively.

Previous research using the PPQ in clinical populations is limited. van den Broek et al. [27] demonstrated that (incentivised) mild traumatic brain injury simulators reported inflated scores on both the clinical and validity scales of the PPQ and could be clearly differentiated from genuine respondents and patients with ABI. Rates of noncredible symptom reporting among chronic pain patients in this study are higher than that previously observed in both healthy and ABI populations completing the PPQ (although lower than study simulators in the original study) [32], lending credence to the idea that people with chronic pain syndromes may be more susceptible to noncredible symptom reporting [35]. Furthermore, although patients with chronic pain more often reported noncredible pain-related than non–pain-related symptoms on the physical validity scale, they also frequently endorsed a wide range of (noncredible) physical symptoms and a comparable number of emotional and cognitive complaints. Patients who crossed the ‘likely noncredible reporting' threshold in one domain tended to do so on one or both other domains, indicating that implausible symptom endorsement in chronic pain reflects a multidimensional response style rather than a domain-specific bias restricted to pain-related phenomena. These findings are also consistent with a recent study which, using a conservative threshold on the Self-Report Symptom Inventory (SRSI), observed notable levels of noncredible symptom presentations (labelled ‘pseudosymptoms') in over a third of patients with fibromyalgia (34%) [36], with patients scoring significantly higher than healthy controls on both motor and cognitive pseudosymptom subscales [36].

While the high prevalence of likely noncredible responding across domains on the PPQ among patients with chronic pain might suggest a tendency to over-report or exaggerate various symptoms, the absence of a definitive criterion for invalid symptom presentation in this population makes any such classification premature. In addition, to the extent, the PPQ may identify implausible symptom endorsement that should not in any event be viewed as synonymous with malingering, as questionnaires necessarily cannot ascertain the respondent's intention, and there is no way to be certain that any over-reporting or implausible symptom presentation is deliberate or deceitful [19]. Other studies have suggested elevated rates of malingering in chronic pain patients where financial incentive is present [14, 15], or observed significantly higher levels of feigned somatic symptoms in disability litigants with musculoskeletal injury relative to pain patients with no evidence of symptom exaggeration [37]. However, the number of patients in this study involved with litigation was negligible and only slightly more than a third were disability benefit recipients. Although higher proportions of these patients reported implausible symptoms in the cognitive (32%) and emotional (48%) domains compared with those not receiving benefits and/or involved in litigation (11% and 24%, respectively), differences were not significant once pain severity was accounted for, suggesting that potential financial incentive is unlikely to explain the overall high levels of noncredible symptom endorsement. It is possible, nevertheless, that this reflects the low number of benefit recipients in the present sample (and loss of power in relevant analyses), rather than an absence of effect. Of course, even in the absence of identifiable external incentives such as potential financial compensation, individuals may be motivated to over-report symptoms for secondary gain (e.g., assistance with work, social security issues or help with accommodation) [17, 18] or to seek treatment [38].

Merckelbach et al. [39] have proposed that over-reporting or implausible symptom presentation is not always intentional and it is an oversimplification to interpret it as always being deliberate and misleading. They suggested that over-reporting may stem from misinformation effects, inattention during the assessment process or be attributable to personality characteristics, such as a tendency to negative affect. Previous research has shown that implausible symptom endorsement can be influenced by an individual's educational level [32, 40]. “Likely noncredible reporting” classification thresholds for the PPQ are adjusted accordingly, mitigating the influence of educational level on implausible self-presentation. It is possible that (genuine) symptoms common to chronic pain syndromes, such as fluctuating states of cognitive dysfunction induced by pain-induced neurophysiological changes and mental health contributors (‘brain fog') [41], could have interfered with respondents' attentive capacity. However, response consistency in the patient group indicates that PPQ responses were unlikely to be adversely affected by gross cognitive difficulties, and it is unclear if any subtle deficit necessarily leads to high rates of noncredible complaints. Nevertheless, in the absence of objective neurocognitive assessment, the present results should be interpreted cautiously.

Consistent with recent findings of greater pain levels reported by those patients with musculoskeletal injury or chronic pain who exhibit somatic amplification [37, 42], patterns of likely noncredible symptom reporting on the PPQ were closely linked with pain severity. Multivariate analyses suggested affective pain severity was more strongly associated with implausible cognitive and emotional symptom reporting and sensory pain severity with implausible physical symptom reporting. In this context, it is worth considering findings of individuals with emotional distress exhibiting elevated scores on other symptom validity instruments including the Structured Inventory of Malingered Symptomatology (SIMS) [39, 43]. For individuals experiencing chronic pain with (external) financial incentives, high levels of self-reported pain catastrophising characterised by a strong tendency to attend to and magnify the threat of pain stimuli have previously been associated with the identification of exaggerated (psychological) symptoms [44]. Together with our findings, these studies suggest that symptom distortion in individuals with chronic pain may be more prevalent in those patients who present with high levels of affective pain, acute somatisation and/or emotional distress.

The relationship between PPQ validity measures and the other SVT and PVT measures indicated that the strongest relationship was between the PPQ and NIM, although this was most apparent in the case of the cognitive and emotional validity scales, rather than physical. This may reflect that the NIM scale of the PAI was primarily developed to assess exaggerated psychopathology, rather than somatic and physical complaints, and, at least by itself, is not sufficiently sensitive to detect MPRD [26]. In contrast, the PPQ validity scales showed limited sensitivity relative to the MSVT, as might be expected bearing in mind that the MSVT is a memory-oriented symptom validity test that measures underperformance and is unlikely to be strongly related to implausible symptom reporting in other domains [45, 46]. The PPQ showed poor sensitivity and specificity relative to the PIM scale. This reflects an expected difference between the PPQ measures and PIM, with the latter measuring defensiveness and favourable impression management, rather than over-reporting [20].

### 4.1. Limitations and Future Studies

There are several limitations to the present study. Although the PPQ has been used successfully to differentiate between simulated brain trauma and neurological patients with acquired brain injuries and controls, with good sensitivity and specificity for the validity scales [27], the psychometric properties of this measure in chronic pain populations have not been established. An assessment of the PPQ's ability to distinguish between genuine responses and simulated chronic pain symptoms in healthy participants is needed. More generally, replicating these findings and checking construct validity and (test–retest) reliability in larger samples of patients would provide evidence of generalisability to other chronic pain populations. Examination of the relationship between the PPQ and other measures with validity scales did support an association between implausible symptom endorsement on the PPQ and exaggerated psychopathology on the PAI, but this was limited by the small number of participants who completed both. Including another (validated) self-report tool specifically intended to detect noncredible symptom presentations, such as the SIMS or the SRSI, could provide a more comprehensive understanding of symptom authenticity. The incorporation of less subjective measures of somatic/pain exaggeration such as functional capacity evaluations (e.g., the Jamar hand dynamometer) may be helpful additions in future work, although the extent to which these types of assessments successfully discriminate submaximal effort from real effort in chronic pain patients is unclear [19, 47].

While the current study illustrated high levels of noncredible symptom endorsement in people experiencing chronic pain, reasons for this warrant further investigation. Including measures that specifically gauge levels of pain catastrophising, somatisation and/or alexithymia would help to establish associations of implausible symptom reporting on the PPQ with these constructs. As noted previously, the role of secondary gain in noncredible symptom presentation is unclear; only 14% of the sample in the present study was in full-time employment while 35% received disability benefits, and motivation to maintain benefits may be relevant or alternatively associated with a concomitant illness identity. More specifically, the utility of the measure in chronic pain patients involved in litigation is unknown and warrants further study. Additionally, patients' analgesic medication use, which has been linked with elevated ratings of pain severity and symptom reporting and increased cognitive complaints in chronic pain populations [48, 49], was not available; their impact on reporting of genuine and implausible symptoms is therefore unknown.

Finally, the cross-sectional study design does not allow causal inferences about observed associations between severity of pain experienced and noncredible symptom presentations across domains in chronic pain patients. A potentially important issue is the predictive validity of symptom validity measures, such as the PPQ, and its utility in treatment planning and evaluation. For example, suboptimal effort on PVTs in veterans undergoing clinically indicated neuropsychological evaluation has been linked with greater utilisation of healthcare clinical services following evaluation [50], while symptom over-reporting in people seeking treatment at an outpatient psychology training clinic has been associated with diminished treatment compliance and premature therapy termination [51, 52]. Significant nonvalid PPQ self-report may be associated with poor treatment engagement, early treatment termination or less favourable clinical outcomes, and an interesting issue for further research will be the extent to which it is predictive of these important clinical issues.

## 5. Conclusions

Detecting invalid symptom response remains challenging, particularly in view of the uncertainty surrounding which are the most appropriate SVTs for assessments, how to best interpret the results from these measures and the lack of consensus on the number of SVT failures required to deem a response set invalid [53]. This is especially complicated for patients with chronic pain given potential risk factors for noncredible symptom presentation, such as somatisation and emotional distress, are themselves strongly implicated in the development of central sensitisation and chronic pain [19]. Furthermore, there is an adverse association between stigma and chronic pain outcomes [54] and questioning the validity of patients' reporting can inadvertently maintain or even increase their reported pain levels and associated functional disability [55, 56]. Nevertheless, the PPQ is a potentially useful tool in the assessment of symptoms in patients with chronic pain, with endorsement of implausible symptoms common across cognitive, emotional and physical domains. Noncredible cognitive and emotional symptom complaints were linked with affective pain severity ratings and NIM as assessed on the PAI, while noncredible physical symptom complaints were associated with the severity of sensory pain. From a clinical perspective, this suggests that patients who exhibit high levels of affective distress linked to their pain may be vulnerable to distorted symptom presentations across domains. Although this likely complicates both the assessment of the nature and severity of a chronic pain patients' difficulties and the subsequent alignment of management goals and tasks with actual needs [57], it should not deprive the patient of an opportunity for appropriate assessment and management of patients at risk. Rather, in the face of noncredible symptom reporting, the most appropriate approach will still inevitably involve the use of a multidisciplinary team focussed on facilitating self-management and improving quality of life despite the presence of persistent pain [19, 58]. Nevertheless, further research is needed to ascertain the extent to which implausible symptom endorsement in patients presenting with chronic pain syndromes is a marker of attentional impairment, reflects somatisation and pain-related psychological distress or is a product of intentional exaggeration, and whether or not it has predictive value to treatment response.

## Figures and Tables

**Table 1:** Demographic and clinical characteristics of patients with chronic pain (*n* = 77).

*Demographic*	
Gender: Female	49 (63.6)
Age (mean (SD; range))	50.5 (12.7; 18–90)
Education	
Low	15 (19.5)
Medium (A-levels, GCSEs)	29 (37.7)
High (degree/diploma)	33 (42.9)
Employment status	
Working (full-/part-time)	27 (35.1)
Student	3 (3.9)
Unemployed	2 (2.6)
Disability benefit	27 (35.1)
Medically retired	13 (16.9)
Retired	5 (6.5)
Litigation involvement	4 (5.2)

*Clinical*	
Pain duration (years; median [IQR])	5.0 (2.8–11.0)
Pain ≥ 3 years	47 (61.0)
Treatment	
Pain clinic	52 (67.5)
Pain management programme	25 (32.5)
Pain severity: SF-MPQ	
Total (0–45; mean (SD))	24.44 (11.26)
Sensory (0–33; mean (SD))	18.49 (8.31)
Affective (0–12; mean (SD))	5.95 (3.74)

*Note:* SF-MPQ data were not available for 4 patients. Numbers represent frequency (percentage) unless otherwise stated.

Abbreviations: IQR = interquartile range, SD = standard deviation, SF-MPQ = short-form McGill Pain Questionnaire.

**Table 2:** Summary statistics for chronic pain participants on the Personal Problem Questionnaire (PPQ) clinical and validity scales (*n* = 75).

**Clinical domain**	**Subscale (0–20)**	**Raw score** **Mean (SD)**	**Range**	***α* (95% CI)**	

Cognitive	Language & Communication	7.12 (4.59)	0–19	0.86 (0.81, 0.90)	
	Memory	9.35 (5.08)	0–19	0.89 (0.85, 0.93)	
	Concentration	9.11 (5.42)	0–20	0.91 (0.87, 0.94)	
	Executive	9.02 (4.13)	0–20	0.78 (0.70, 0.85)	

Emotion	Anger	8.08 (5.26)	0–20	0.91 (0.87, 0.94)	
	Anxiety	8.62 (5.38)	0–20	0.91 (0.87, 0.93)	
	Depression	10.00 (5.09)	0–20	0.88 (0.83, 0.92)	
	Stress	9.27 (5.09)	0–20	0.88 (0.84, 0.92)	

Physical	Pain	15.01 (3.74)	4–20	0.75 (0.65, 0.83)	
	Neurological	9.41 (4.37)	0–18	0.80 (0.72, 0.86)	
	Disability & Restrictions	8.53 (4.85)	0–20	0.83 (0.77, 0.88)	
	Somatic	9.07 (4.33)	1–19	0.76 (0.67, 0.84)	

**Scale totals (0–80)**					***T*-score** **Mean (SD)**

Cognitive		34.60 (17.84)	1–78	0.96 (0.95, 0.97)	64.49 (13.05)
Emotional		35.92 (18.45)	0–79	0.96 (0.95, 0.97)	65.13 (13.24)
Physical		41.89 (14.43)	9–74	0.91 (0.88, 0.94)	77.41 (10.97)

**Validity scale totals (0–24)**					**Likely noncredible** ***n* (%)**

Cognitive		3.16 (4.01)	0–23	0.86 (0.80, 0.90)	14 (19.2)
Emotional		3.96 (4.87)	0–24	0.88 (0.83, 0.92)	24 (33.3)
Physical		4.08 (4.38)	0–24	0.86 (0.80, 0.90)	26 (35.6)

*Note:* In the few cases (< 1%) where a participant failed to respond to one item on a subscale, the total subscale score was calculated by imputing the mean item score for the missing item; α = Cronbach's alpha; CI = confidence interval (calculated using Feldt's formula); Cronbach's alphas were calculated using data from participants who provided responses to all items on the relevant subscale.

**Table 3:** Associations of Personal Problem Questionnaire (PPQ) clinical scale *T*-scores with pain severity and disability benefit/litigation status for chronic pain participants (*n* = 75).

**Cognitive**				

*Pain*	*r/rho*	*p*	*B (95% CI)*	*p*
Duration	0.05	0.682		
Sensory	**0.42**	**< 0.001**	0.20 (−0.28, 0.75)	0.442
Affective	**0.47**	**< 0.001**	**1.13 (0.19, 1.96)**	**0.017**

*Disability status*	*Mean (SD)*	*p*		
Not on benefits/litigation (*n* = 47)	**61.09 (13.63)**			
Benefits/litigation (*n* = 28)	**70.21 (9.79)**	**0.001**	**6.47 (1.56, 10.95)**	**0.013**

**Emotional**				

*Pain*	*r/rho*	*p*	*B (95% CI)*	*p*
Duration	−0.02	0.875		
Sensory	**0.52**	**< 0.001**	0.37 (−0.06, 0.79)	0.094
Affective	**0.56**	**< 0.001**	**1.34 (0.39, 2.30)**	**0.006**

*Disability status*	*Mean (SD)*	*p*		
Not on benefits/no litigation (*n* = 47)	**62.35 (14.06)**			
Benefits/litigation (*n* = 27)	**69.95 (10.23)**	**0.017**	3.28 (−2.23, 8.77)	0.243

**Physical**				

*Pain*	*r/rho*	*p*	*B (95% CI)*	*p*
Duration	−0.01	0.915		
Sensory	**0.56**	**< 0.001**	**0.40 (0.05, 0.75)**	**0.024**
Affective	**0.55**	**< 0.001**	**0.90 (0.13, 1.66)**	**0.022**

*Disability status*	*Mean (SD)*	*p*		
Not on benefits/no litigation (*n* = 47)	**74.96 (12.03)**			
Benefits/litigation (*n* = 27)	**81.65 (7.26)**	**0.011**	3.39 (−1.07, 7.84)	0.136

*Note:* In the few cases where a participant failed to respond to one item on a subscale, the total subscale score was calculated by imputing the mean item score for the missing item. In one case, on each of the emotional and physical clinical scales, more than one item response was missing and no summary scores were calculated. Sensory and affective pain severity scores (from SF-MPQ) were missing for 4 patients. Only variables showing significant associations (i.e., *p* < 0.05) in univariate analyses (Pearson r/Spearman rho and independent group *t*-tests) were included in a generalised linear model (GLM) with linear model type. Magnitudes of effects in GLM were described by (unstandardised) beta (B) values with 95% confidence intervals (CIs). Reference categories were ‘Not on benefits/no litigation' for disability status. Significant effects are highlighted in bold.

**Table 4:** Associations of valid/invalid responding on the Personal Problem Questionnaire (PPQ) with pain severity and disability benefit/litigation status for chronic pain participants.

**Cognitive**	**Valid** **(*n* = 59)**	**Invalid** **(*n* = 14)**			

*Pain*	*Mean (SD)*	*Mean (SD)*	*p*	*OR (95% CI)*	*p*
Duration	7.81 (8.78)	14.14 (16.21)	0.198		
Sensory	**17.27 (8.34)**	**23.00 (6.72)**	**0.024**	0.98 (0.86, 1.11)	0.715
Affective	**5.20 (3.52)**	**8.92 (3.28)**	**< 0.001**	**1.34 (1.01, 1.79)**	**0.042**

*Disability status*	*n (%)*	*n (%)*	*p*		
Not on benefits/no litigation (n = 45)	**40 (88.9)**	**5 (11.1)**			
Benefits/litigation (*n* = 28)	**19 (67.9)**	**9 (32.1)**	**0.035**	2.81 (0.68, 11.64)	0.155

**Emotional**	**Valid** **(*n* = 48)**	**Invalid** **(*n* = 24)**			

*Pain*	*Mean (SD)*	*Mean (SD)*	*p*	*OR (95% CI)*	*p*
Duration	8.19 (8.69)	10.94 (14.20)	0.404		
Sensory	**15.82 (7.70)**	**23.17 (7.62)**	**< 0.001**	1.06 (0.95, 1.17)	0.291
Affective	**4.62 (3.33)**	**8.30 (3.42)**	**< 0.001**	**1.26 (1.01, 1.58)**	**0.041**

*Disability status*	*n (%)*	*n (%)*	*p*		
Not on benefits/no litigation (*n* = 45)	**34 (75.6)**	**11 (24.4)**			
Benefits/litigation (*n* = 27)	**14 (51.9)**	**13 (48.1)**	**0.039**	2.06 (0.61, 6.89)	0.242

**Physical**	**Valid** **(*n* = 47)**	**Invalid** **(*n* = 26)**			

*Pain*	*Mean (SD)*	*Mean (SD)*	*p*	*OR (95% CI)*	*p*
Duration	8.09 (8.75)	9.63 (12.98)	0.605		
Sensory	**15.45 (7.48)**	**23.80 (6.96)**	**< 0.001**	**1.12 (1.01, 1.24)**	**0.028**
Affective	**4.77 (3.54)**	**8.16 (2.90)**	**< 0.001**	1.15 (0.93, 1.43)	0.191

*Disability status*	*n (%)*	*n (%)*	*p*		
Not on benefits/no litigation (*n* = 46)	32 (69.6)	14 (30.4)			
Benefits/litigation (*n* = 27)	15 (55.6)	12 (44.4)	0.228		

*Note:* In the few cases where a participant failed to respond to one item on a subscale, the total subscale score was calculated by imputing the mean item score for the missing item. In two cases on each of the cognitive and physical validity scales and one case on the emotional validity scale, more than one item response was missing and no summary scores (and valid/invalid classifications) were calculated. Sensory and affective pain severity scores (from SF-MPQ) were missing for 4 patients. Only variables showing significant associations (i.e., *p* < 0.05) in univariate analyses (chi-square and independent group *t*-tests) were included in a generalised linear model (GLM) with logistic model type. Magnitudes of effects in GLM were described by odds ratios (OR) indicating change in odds of invalid responding (scoring ≥ cut-off) per 1 unit increase on relevant measure with 95% confidence intervals (CIs). Reference categories were ‘Not on benefits/no litigation' for disability status. Where univariate analyses indicated significant effects of age (emotional validity scale) or the quadratic function of age (cognitive validity scale), these terms were entered in corresponding GLM models (coefficients not presented). Significant effects are highlighted in bold.

**Table 5:** Sensitivity and specificity of the Personal Problem Questionnaire (PPQ) validity scales in the chronic pain sample (*n* = 75) with reference to pass/failure on the Medical Symptom Validity Test (MSVT) and negative/positive impression management subscale of the Personality Assessment Inventory (PAI).

**Clinical domain**		**MSVT**			**Negative impression management**			**Positive impression management**		
		**Fail**	**Pass**		**Moderate/severe exaggeration**	**No exaggeration**		**Moderate/severe exaggeration**	**No exaggeration**	

PPQ Cognitive	Nonvalid	3	11	PPP 0.21	7	1	PPP 0.88	1	7	PPP 0.13
	Valid	4	54	NPP 0.93	3	14	NPP 0.82	7	10	NPP 0.59
		Sensitivity 0.43	Specificity 0.83		Sensitivity 0.70	Specificity 0.93		Sensitivity 0.13	Specificity 0.59	

PPQ Emotional	Nonvalid	3	21	PPP 0.13	9	1	PPP 0.90	1	9	PPP 0.10
	Valid	4	43	NPP 0.91	1	13	NPP 0.93	6	8	NPP 0.57
		Sensitivity 0.43	Specificity 0.67		Sensitivity 0.90	Specificity 0.93		Sensitivity 0.14	Specificity 0.47	

PPQ Physical	Nonvalid	5	21	PPP 0.19	7	3	PPP 0.70	3	7	PPP 0.30
	Valid	2	44	NPP 0.96	3	12	NPP 0.80	6	9	NPP 0.60
		Sensitivity 0.71	Specificity 0.68		Sensitivity 0.70	Specificity 0.80		Sensitivity 0.33	Specificity 0.56	

Abbreviations: NPP = negative predictive power, PPP = positive predictive power.

## Data Availability

The data that support the findings of this study are available upon request from the corresponding author. The data are not publicly available due to privacy or ethical restrictions.
